# Metagenome Analysis of the Bacterial Characteristics in Invasive *Klebsiella Pneumoniae* Liver Abscesses

**DOI:** 10.3389/fcimb.2022.812542

**Published:** 2022-07-15

**Authors:** Zhijie Zhang, Hairui Wang, Yawen Guo, Zhaoyu Liu, Zhihui Chang

**Affiliations:** ^1^ Department of Laboratory Medicine, Shengjing Hospital of China Medical University, Shenyang, China; ^2^ Department of Radiology, Shengjing Hospital of China Medical University, Shenyang, China

**Keywords:** *Klebsiella pneumoniae*, liver abscess, metagenome, metabolic pathway, invasiveness

## Abstract

**Background:**

*Klebsiella pneumoniae* liver abscess (KPLA) combined with extrahepatic migratory infection (EMI) is defined as invasive KPLA (IKPLA) and is associated with a poor prognosis. The mechanism of IKPLA formation is yet to be elucidated. In this study, metagenomic sequencing was used to compare the bacterial characteristics between IKPLA and KPLA to explore the underlying mechanism of invasiveness.

**Methods:**

Clinical details, imaging, and microbial features were retrospectively evaluated by medical record review. Metagenomic sequencing was performed on the pus samples of liver abscesses whose culture results were indicative of monomicrobial *Klebsiella pneumoniae* (*K. pneumoniae*). Bacterial diversity and composition in IKPLA and KPLA were comparatively analyzed, and the key pathways and genes that may affect invasiveness were further explored.

**Results:**

Sixteen patients were included in this study. Five patients with EMI were included in the IKPLA group, and the other eleven patients without EMI were assigned to the KPLA group. There was no statistical difference in the hypermucoviscous phenotype and serotype of *K. pneumoniae* between the two groups. The bacterial diversity of IKPLA was lower than that of KPLA. The abundant taxa in the IKPLA group were primarily species of *unclassified Enterobacteriaceae* and *K. pneumoniae*. The KPLA group had a high abundance of the genera *Tetrasphaera* and *Leuconostoc*. Metabolic pathway genes represented most of the enriched genes in IKPLA. Fourteen pathogenic genes with significant differences in abundance were identified between the two groups, including ybtS, fepC, phoQ, acrB, fimK, magA, entC, arnT, iucA, fepG, oqxB, entA, tonB, and entF (p < 0.001).

**Conclusion:**

The diversity and bacterial composition of IKPLA were significantly different from those of KPLA. Microbiological changes in the abscess, activation of the related metabolic pathways, and the pathogenic gene expression may constitute a novel mechanism that regulates the invasiveness of KPLA.

## Introduction

*Klebsiella pneumoniae (K. pneumoniae)* is an emerging cause of community-acquired liver abscess, approximately 20% of which results in extrahepatic migratory infection (EMI) and is defined as invasive *K. pneumoniae* liver abscesses (IKPLA). EMI is a condition wherein *K. pneumoniae* in the liver abscess metastasize to distant sites, most commonly to the eyes, lungs, and central nervous system (Chung et al., 2007; Siu et al., 2012; Shin et al., 2013; Guo et al., 2017). During the past two decades, KPLA has been increasingly reported in Asia, and this invasive syndrome is emerging as a global concern (Chung et al., 2007; Siu et al., 2012; Guo et al., 2017; Sun et al., 2021). Catastrophic disability caused by ocular or neurological complications arising from IKPLA can lead to a poor long-term prognosis (Lin et al., 2012). The invasive mechanism of *K. pneumoniae* includes the hypermucoviscous phenotype associated with serotypes K1 and K2 and the expression of virulence genes (Lin et al., 2004; Fang et al., 2007; Yeh et al., 2010; Siu et al., 2012). However, invasiveness has not been fully explained from the perspective of hypermucoviscous phenotype, serotype, or virulence genes of *K. pneumoniae* (Siu et al., 2012).

In recent years, increasing number of studies have been conducted on the etiology of liver abscesses by using microbiomic methods (Song et al., 2014; Reyna-Fabian et al., 2016; Chen et al., 2018; Chen et al., 2019; Kim et al., 2020; Bivand et al., 2021; Molton et al., 2021). The bacterial diversity and composition in the liver abscesses can be obtained using sequencing technology, and species that cannot be detected by conventional bacterial culture can also be discovered (Song et al., 2014; Chen et al., 2018; Bivand et al., 2021). One study employed 16S rRNA gene sequencing and showed that amebic liver abscess can be coinfected by bacteria and that bacteria could increase the virulence of ameba (Reyna-Fabian et al., 2016). This finding suggests that the bacterial composition of an abscess may affect the virulence of pathogenic bacteria. However, there are few reports focusing on the bacterial diversity and composition in KPLA.

Whole-genome analysis suggests four members of the *K. pneumoniae* species complex (KpSC), namely, *K. pneumoniae*, *K. quasipneumonia*, *K. variicola*, and *K. quasivariicola*, as distinct pathogenic species (Holt et al., 2015). Relatively little is known about the difference in the population structure of KpSC between IKPLA and KPLA. Many consider this knowledge to be fundamental to support the efforts to explore the underlying mechanism of KPLA invasiveness.

Hence, we performed metagenomic sequencing of pus from patients with KPLA. The bacterial diversity and composition as well as the population structure of KpSC between the groups were analyzed to establish the underlying mechanism of invasiveness.

## Materials and Methods

### Ethics Statement

This study was approved by the ethics committee of Shengjing Hospital of China Medical University (No. 2015PS184K); written informed consent was obtained from all the participants. The data for this study were from a prospective registry study predicting the risk of migratory infection in KPLA with registration number ChiCTR-ROC-15006581.

### Patients and Clinical Samples

Diagnostic criteria for liver abscess were the same as in previous studies (Lee et al., 2008; Lai and Lin, 2010; Alsaif et al., 2011; Chan et al., 2013; Chang et al., 2015). The criteria were chiefly based on clinical manifestations, laboratory examination, and imaging features. The pus of all patients who underwent puncture drainage for liver abscess was collected prospectively from October 2019 to December 2019.

We routinely perform percutaneous drainage and antibiotic treatment for patients with a liver abscess, which has a maximum diameter that is greater than 3 cm. All patients were started on antibiotics prior to percutaneous abscess drainage. The method of percutaneous drainage was the same as that employed in our previous study (Wang et al., 2021). The pus was aspirated with a sterile syringe after a successful puncture and divided into two samples. One sample was used for bacterial culture, and the other was routinely stored in a −80°C biobank archive to be later retrieved for metagenomic analysis. Before obtaining the samples, no contrast agent was instilled into the abscess cavity to reduce the effect of the contrast agent on bacterial activity. Finally, the pus samples that yielded bacterial culture results of monomicrobial *K. pneumoniae* were analyzed by metagenomic sequencing.

### Culture

The pus samples were both gram stained and grown on relevant growth medium under aerobic and anaerobic conditions as per Clinical Laboratory Standards Institute guidelines. All bacterial isolates were identified using matrix-assisted laser desorption/ionization-time of flight mass spectrometry (Microflex, Bruker Biotyper, Bremen, Germany).

### String Test for Hypermucoviscosity

The string test was performed as previously described to determine bacterial hypermucoviscosity (Fang et al., 2004). Briefly, a bacteriologic loop was used to stretch a mucoviscous string from a second subculture of *K. pneumoniae* colony on blood agar (Sigma–Aldrich). Hypermucoviscosity is semi-quantitatively defined by the formation of viscous strings > 5 mm in length.

### Polymerase Chain Reaction (PCR) for Genotyping

K1, K2, K5, K20, K54, and K57 serotypes were identified by detection of K serotype-specific wzy and wzx alleles using the PCR method as previously described (Fang et al., 2007; Turton et al., 2010). The PCR products were visualized using 1% agarose gel electrophoresis and sequenced commercially. The basic local alignment search tool (BLAST) program was used for final serotype identification.

### DNA Extraction and High-Throughput Sequencing analysis

DNA was isolated from the pus samples using the High Pure PCR Template Preparation Kit (Roche, Mannheim, Germany). DNA samples were subjected to high-throughput sequencing on the Illumina NovaSeq 6000 platform with PE150bp in paired-end mode. Sequence reads were assembled using the *de novo* approach with IDBA-UD. We used MetaGeneMark to predict the open reading frames (ORFs) of each sample and mixed assembled contigs (≥500bp) and filter the sequences with an ORF length of <100bp according to the prediction results. The total length of the predicted ORFs was 4.3 Mbp. Among these ORFs, 3445 (74.12%) were complete genes. Based on the ORF prediction results, CD-HIT software was used to remove the redundant genes. Simultaneously, the longest sequence was selected as the representative sequence by clustering with 95% identity and 90% coverage. We compared the clean data of each sample with the gene sequence using Bowtie2 and calculated the number of reads of each gene in each sample. We then filtered out the genes with ≤2 reads in all samples to obtain the unigenes used for further analysis.

### Taxonomy Prediction

The unigene sequences were BLASTed against the nonredundant database using DIAMOND software. After comparison, we selected the best result for species classification. The information of each taxonomical hierarchy (phylum, class, order, family, genus, and species) was obtained.

### Population Structure of KpSC

To explore the difference in the population structure of KpSC between IKPLA and KPLA, the abundance of the four members of KpSC was compared using one-way analysis of variance.

### Functional Annotations

To obtain functional information of unigenes, their sequences were BLASTed against the Kyoto Encyclopedia of Genes and Genomes (KEGG) database using DIAMOND software. The annotation of KEGG alignment sequences (hits) with the highest score was taken as the annotation of the unigene sequence. Combined with the hierarchical structure of the KEGG pathway database, we can record each level of the KEGG pathway. KEGG pathway enrichments were also performed. The DIAMOND software was also used to annotate the unigene comparison to the Pathogen Host Interactions (PHI) database, and the annotation results were further screened to identify the potentially pathogenic genes in IKPLA with the following parameters: minimum identity 90% and E value ≤1e-5. PHI-base contains molecular and biological information on genes that had been shown to affect the outcome of host–pathogen interactions (Urban et al., 2020).

### Data Analysis

Alpha diversity was measured using the Chao1 and Shannon diversity indices. Beta diversity was measured using principal coordinate analysis (PCoA) and was based on the Bray–Curtis distance. Analysis of similarities (ANOSIM) was performed to detect significant dissimilarity or similarity in the community composition between IKPLA and KPLA. Differential taxa abundance and KEGG modules were tested with the Wilcoxon rank-sum test. Linear discriminant effect size (LEfSe) analysis was performed to determine which taxa were significantly different between the groups (linear discriminant analysis LDA ≥ 4, p < 0.05). The top 20 species and the top 20 KEGG modules were selected according to their average relative abundances, and the correlation between them was determined using Spearman correlation analysis. Clustering correlation heatmap analysis with signs was performed using the OmicStudio tools. Data analysis was performed using R version 3.6.1, with statistical significance set at p < 0.05.

## Results

### Clinical Features of the Patients

Sixteen patients diagnosed with KPLA were included in this study. A detailed flow chart of patient inclusion is provided in **Supplementary Figure 1**. The age of the patients ranged from 30 to 80 years. Five patients with EMI (four patients combined with septic pulmonary embolism and one patient combined with septic pulmonary embolism and endophthalmitis) were included in the IKPLA group. The other 11 cases were assigned to the KPLA group. The basic data and clinical and computed tomography features of the two groups are listed in **Table 1**. The proportion of men in the KPLA group was significantly higher than that in the IKPLA group (p = 0.036). Platelets were significantly lower in the IKPLA group than in the KPLA group (p = 0.002). Thrombophlebitis of the hepatic veins was common in the IKPLA group (p = 0.003).

**Table 1:** Comparison of clinical, computed tomography, and microbial characteristics between KPLA and IKPLA.

	KPLA (n = 11)	IKPLA (n = 5)	P
Age*	64 (36-78)	66 (30-80)	0.997
Male	9 (81.8%)	1 (20.0%)	0.036
Underlying diseases			
Diabetes	6 (54.5%)	4 (80.0%)	0.588
Biliary disease	3 (27.3%)	0 (0.0%)	0.509
Digestive tumor	2 (18.2%)	0 (0.0%)	1
Laboratory examination			
White blood cell (10^9^/L)	11.2 (5.2-20.6)	11.0 (1.9-20.4)	0.955
Neutrophils (10^9^/L)	7.9 (2.6-18.9)	13.9 (6.3-53.5)	0.061
Platelets (10^9^/L)	305 (103-581)	30 (11-100)	0.002
Hemoglobin (g/L)	120 (80 -142)	103 (67-139)	0.532
Prothrombin time(s)	14.0 (11.0-15.5)	14.3 (12.6-15.6)	0.532
C-reactive protein (mg/L)	143.0 (67.7-277.0)	147.0 (58.3-271.0)	0.758
Albumin (g/L)	30.6 (24.9-39.6)	24.2 (22.7-33.1)	0.126
Total bilirubin (µmol/L)	16.5 (5.1-69.9)	15.6 (10.3-39.4)	0.156
Creatinine (µmol/L)	53.4 (43.4-79.3)	73.0 (55.3-112.1)	0.015
Fasting blood glucose (mmol/L)	10.0 (4.5-16.8)	10.4 (6.9-22.1)	0.462
CT features			
Maximal diameter* (cm)	58 (13-145)	56 (44-110)	0.779
Multiple	2 (18.2%)	3 (60.0%)	0.245
Septation	6 (54.5%)	4 (80.0%)	0.588
Gas	1 (9.1%)	1 (20.0%)	1
Thrombophlebitis	0 (0.0%)	4 (80.0%)	0.003
ICU admission	0 (0.0%)	1 (20.0%)	0.313
In-hospital mortality	0 (0.0%)	1 (20.0%)	0.313
Serotype			
K1	8 (72.7%)	3 (60.0%)	1.0
K2	1 (9.1%)	0 (0.0%)	1.0
Non-K1K2	2 (18.2%)	2 (40.0%)	0.547
Hypermucoviscous	9 (81.8%)	4 (80.0%)	1

*Median (Minimum - Maximum); CT, Computed tomography; ICU, Intensive Care Unit.

### Genotyping and Hypermucoviscous Phenotype

**Table 1** shows the genotype distribution of the 16 strains. Serotype detection was performed in all strains. Hypermucoviscous phenotype was detected in 81.25% (13/16) of the *K. pneumoniae* strains. There was no statistical difference in the serotype of *K. pneumoniae* and hypermucoviscous phenotype between the two groups.

### Genomes Assembled from the Abscess Microbiome

**Figure 1:**
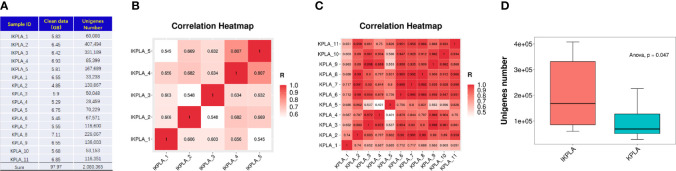
Comparison of unigenes identified between the two groups. **(A)** The sequencing analysis found a total of 97.97-giga base (Gb) reads of high-quality sequences from the microbial DNA of pus samples. Using the CD-HIT program, 2,080,365 unigenes were assembled as the basis of taxonomy and functional annotation. **(B, C)** Analysis of the genome across different samples showed a high correlation of gene profiles in both groups. The color from red to white represents the correlation coefficient from high to low (from 1 to 0). **(D)** The sum of the unigene numbers of IKPLA was greater than that of KPLA (p = 0.047).

As shown in **Figure 1A**, the sequencing analysis found a total of 97.97-gigabase (Gb) reads of high-quality sequences (average of 6.12 Gb per sample) from the microbial DNA of the pus samples. In all the 16 samples, the average GC percentage was 57.29%. Using the CD-HIT program, 2,080,365 unigenes were assembled as the basis of taxonomy and functional annotation. Correlation of the genome across different samples showed the acceptable similarity of gene profiles in both groups (**Figures 1B, C**). The sum of the unigene numbers of the IKPLA was higher than that of the KPLA (**Figure 1D**).

### Comparison of Microbiota Diversity Between KPLA and IKPLA

**Figure 2:**
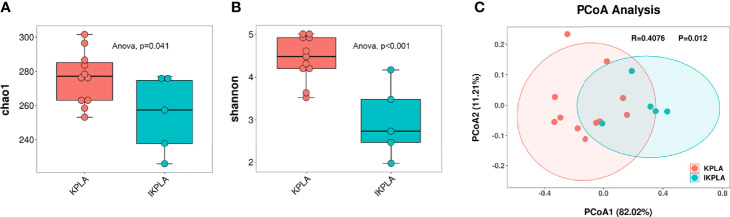
Alpha and beta diversity plots to examine the differences between the groups. Both the Chao1 and Shannon indices in the IKPLA group were lower than those in the KPLA group **(A, B)**. PCoA analysis based on log transformed metagenomic species abundances **(C)**. Associated P values < 0.05 were considered as significant separation between KPLA and IKPLA.

To determine if there were differences between the groups, microbial diversity and composition were examined. The alpha diversity analysis was conducted using two indexes, namely the Chao1 index indicating community richness and the Shannon index indicating community diversity. In the IKPLA group, both the indexes were lower than those in the KPLA group (**Figures 2A, B**).

To investigate the similarity of the microbiota in each individual among the two groups, we used PCoA based on the Bray–Curtis distance of the relative abundance of species to visualize the distribution and clustering of the subjects. These two groups clustered separately from each other (**Figure 2C**), as supported by the analysis of similarities (ANOSIM) test (R = 0.4076, p = 0.012).

### Identification of Significant Taxa Differences

**Figure 3:**
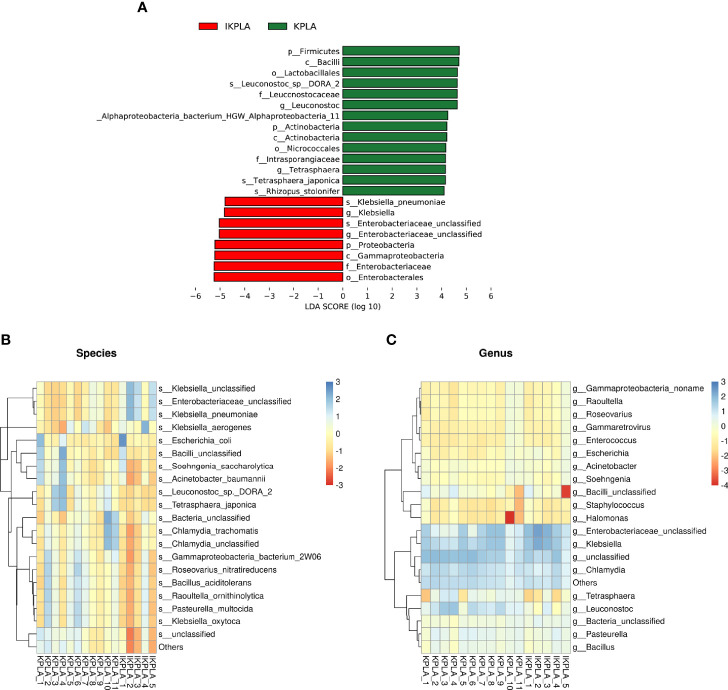
Linear discriminant-analysis effect-size (LEfSe) analyses to identify the taxonomic features most likely to explain the differences between the groups. A total of 22 taxa were identified as significantly different between KPLA and IKPLA by LEfSe **(A)**. Clustering correlation heatmaps based on the top 20 genus or species showed that there were differences in the species level **(B)** but no difference in the genus level **(C)**.

To identify meaningful differences in specific taxonomic groups, LEfSe was used to identity taxa with significant differences between the groups (LDA ≥ 4; p < 0.05). A total of 22 taxa were identified to be significant for KPLA versus IKPLA. The taxa that were abundant in the IKPLA group primarily belonged to species of *unclassified Enterobacteriaceae, and K. pneumoniae*. The KPLA group had a higher abundance of the genera *Tetrasphaera* and *Leuconostoc*, and species of *Alphaproteobacteria_bacterium_HGW_Alphaproteobacteria_11, Rhizopus_stolonifer, Tetrasphaera_japonica, and Leuconostoc_sp:DORA_2.* Other differential taxa in the phylum, class, order, and family levels are shown in **Figure 3A**. Clustering correlation heatmaps based on the top 20 genus or species showed the presence of differences at the species level (**Figure 3B**) but no difference at the genus level (**Figure 3C**).

### Population Structure of *K. Pneumoniae*

**Figure 4:**
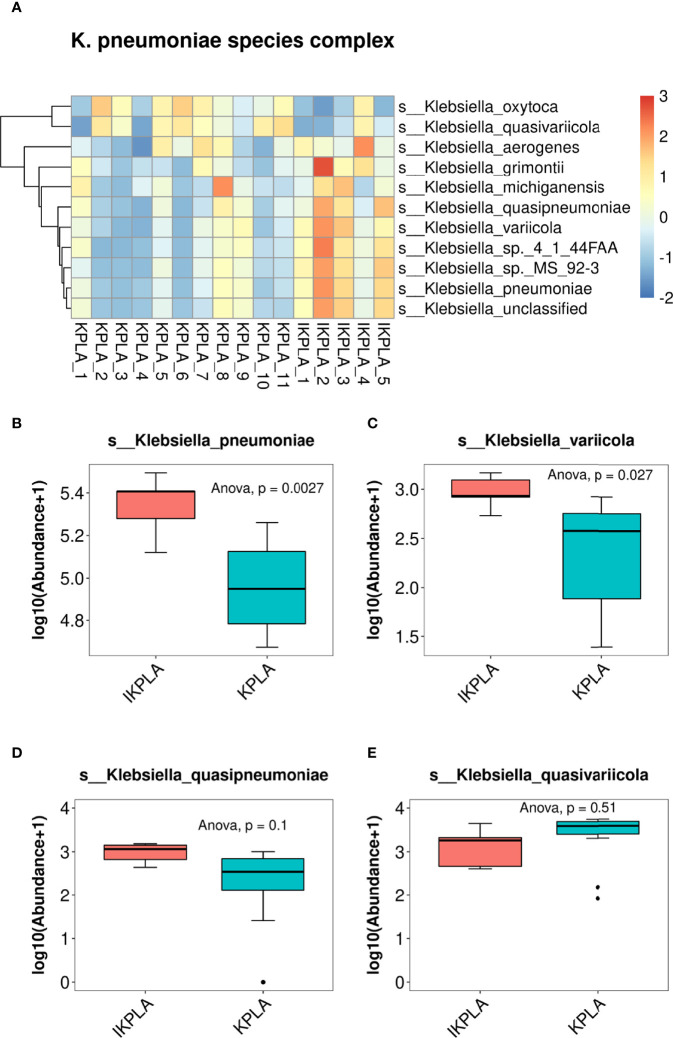
Population structure of *K. pneumoniae*. The clustering correlation heatmap showed that there were differences in the abundance of many species of *Klebsiella* between KPLA and IKPLA **(A)**. Furthermore, comparative analysis was performed for four members of the *Klebsiella pneumoniae* species complex (KpSC). The results showed that the abundances of *K. pneumonia* **(B)** and *K. variicola* **(C)** were significantly different between the two groups, but there was no difference for *K. quasipneumoniae* **(D)**, and *K. quasivariicola* **(E)**.

First, we compared the abundance of all species included in the genus *Klebsiella*. Clustering correlation heatmaps showed that there were differences in the abundance of many species in the two groups (**Figure 4A**). Furthermore, comparative analysis was performed for four members of the KpSC, including *K. pneumoniae*, *K. quasipneumoniae*, *K. variicola*, and *K. quasivariicola*. The results showed that the abundances of *K. pneumoniae* and *K. variicola* were significantly different between the two groups, but there was no difference for *K. quasipneumoniae* and *K. quasivariicola* (**Figures 4B–E**).

### Functional Characteristics of the Microbiota

To evaluate the functional potential of the microbiota between KPLA and IKPLA, unigene BLAST was conducted using the KEGG Orthology (KO) database. In this study, 1,201 KO nesting sites were identified **(** **Supplementary Table 1** **)**. We further mapped these KOs to KEGG modules and pathways. Five main pathways in the KEGG modules were identified, and the most abundant one was the metabolism pathway (**Supplementary Figure 2**).

**Figure 5:**
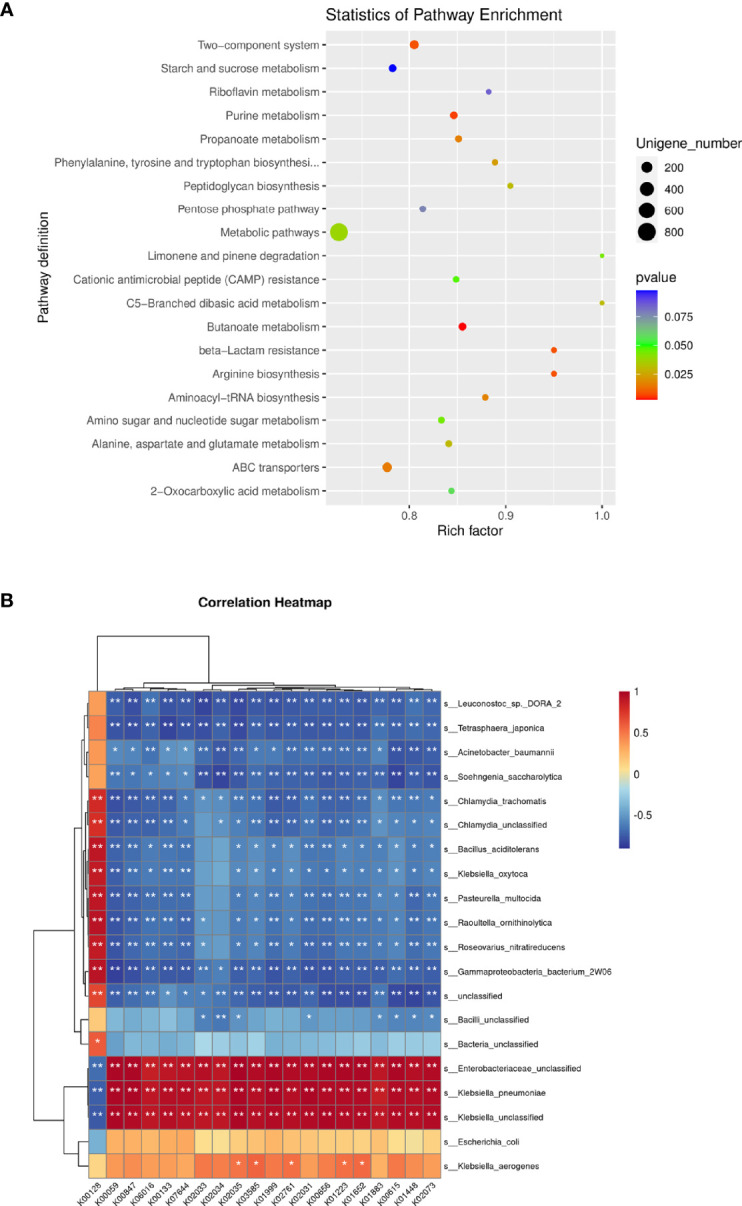
Functional Characteristics of the Microbiota. Fifteen statistically significant pathways were enriched in the IKPLA group. **(A)** Among these pathways, the metabolic pathways represented most of the enriched genes. **(B)** The correlation between the top 20 species and the top 20 KOs was illustrated using clustering correlation heatmap. Positive and negative correlations are shown as red and blue in the heat map, respectively. ∗ Indicates p < 0.05, ∗∗ Indicates p < 0.01.

Specifically, the metabolic pathways of butanoate metabolism, purine metabolism, beta-lactam resistance, arginine biosynthesis, two-component system, ATP-binding cassette (ABC) transporters, propanoate metabolism, aminoacyl-tRNA biosynthesis, phenylalanine, tyrosine, and tryptophan biosynthesis, alanine, aspartate, and glutamate metabolism, peptidoglycan biosynthesis, C5-branched dibasic acid metabolism, limonene and pinene degradation, and amino sugar and nucleotide sugar metabolism were enriched in the IKPLA group. Among these pathways, the metabolic pathway genes represented most of the enriched genes (15.48%) (**Figure 5A**).

The correlation between the top 20 species and the top 20 KOs was shown by a clustering correlation heatmap (**Figure 5B**). Except for k00128 (pyruvate metabolism), all KOs were positively correlated with *K. pneumoniae*.

### Key Genes Annotated in PHI-Base

**Figure 6:**
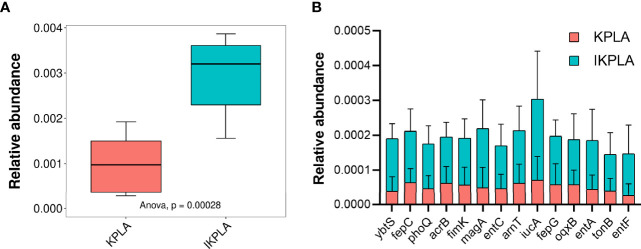
The composition of pathogenic genes in KPLA and IKPLA groups. **(A)** The relative abundance of PHI genes of *K. pneumonia* in the KPLA and IKPLA groups. **(B)** 14 pathogenic genes with significant differences in abundance were identified between the two groups, including ybtS, fepC, phoQ, acrB, fimK, magA, entC, arnT, iucA, fepG, oqxB, entA, tonB, and entF (p < 0.001).

Forty key genes from *K. pneumoniae* were annotated using PHI-base as shown in **Supplementary Table 2**. We investigated the enrichment of pathogenic genes in different groups. The statistical results indicated that the relative abundance of PHI genes in IKPLA was significantly higher than that of the KPLA group (**Figure 6A**). Overall, 14 pathogenic genes with significant differences in relative abundance were identified between the two groups (p < 0.001, **Figure 6B**).

## Discussion

Our results strongly suggest that KPLAs are mixed abscesses (*K. pneumoniae* with other bacteria). Indeed, the mean abundance ratio of *Klebsiella* at the genus level was only 18.5% and the ratio of *K. pneumonia* at the species level was only 14.0% in all KPLAs. KPLA infections originate from the intestinal flora (Fung et al., 2012). The strains were found to cross the intestinal barrier and pass *via* the portal vein to the liver in an animal model (Tu et al., 2009). Hence, we hypothesized that some intestinal flora accompanied *K. pneumoniae* into the liver during the formation of KPLA. One study has shown that *E. histolytica* trophozoites and intestinal bacteria can exist simultaneously in ameba liver abscesses (Reyna-Fabian et al., 2016), which supports our hypothesis. Considering the restricted clinical culture conditions at present, traditional culture media may not be suitable for the growth of some pathogenic bacteria.

One of our major findings was a high proportion of *unclassified Enterobacteriaceae* and *K. pneumoniae* in IKPLAs. On the other hand, the KPLA group had a great abundance of the genera *Tetrasphaera* and *Leuconostoc*. The genus *Tetrasphaera* is involved in enhanced biological phosphorus removal (Nielsen et al., 2019). *Tetrasphaer*a may be able to influence the biological behavior of *K. pneumoniae* by regulating the total amount of phosphorus (Sugeçti, 2021). One study reported that *Leuconostoc lactis* can cause liver abscess formation (Vagiakou-Voudris et al., 2002). Although the interaction between *K. pneumoniae* and *Tetrasphaera* or *Leuconostoc* is unclear, several studies have confirmed that bacterial interaction can affect the virulence of pathogenic bacteria (Zhang et al., 2020). Based on this observation, we speculated that *K. pneumoniae* may gain or lose its high virulence phenotype during interaction with other bacteria in the microenvironment of KPLAs. Hence, it is necessary to ascertain the impact of coexisting bacteria on the invasiveness of *K. pneumoniae*.

We also observed the relative abundance of four members of the KpSC, including *K. pneumoniae*, *K. quasipneumoniae*, *K. variicola*, and *K. quasivariicola*. The results showed that patients with IKPLA had more *K. pneumoniae* and *K. variicola* in their pus than those with KPLA. Of late, *K. variicola*, as a novel bacterial species, is gaining recognition as a cause of several human infections and is associated with gas formation (Jie et al., 2021). Gas formation is common in KPLA and is associated with poor outcomes (Thng et al., 2018). Because only two patients in this study presented with gas formation, the relationship between gas formation and the abundance of. *K. variicola* was not elucidated. *K. variicola* exhibits four major virulence factors, namely, capsule, lipopolysaccharide, siderophores, and fimbriae. It has been suggested that *K. variicola* can be as virulent as *K. pneumoniae* (Maatallah et al., 2014; Long et al., 2017; Rodríguez-Medina et al., 2019). This virulence implies that the invasiveness of KPLA may be caused by both *K. pneumoniae* and *K. variicola*.

Our study revealed significant changes in the abundance of unigenes belonging to metabolic pathways that are known or predicted to be involved in the invasiveness of KPLA. Many studies have also shown that the metabolic pathway play an important role in the virulence and drug resistance of *K. pneumoniae* (Cesur et al., 2019; Russo and Gulick, 2019; Silver et al., 2019; Tang et al., 2020; Xie et al., 2020; Zhao and Tian, 2021). Additionally, multiple pathways differed between the two groups. Among them, the ABC -transporter-dependent pathways are widespread among gram-negative bacteria (Greenfield and Whitfield, 2012). The two-component system present in *K. pneumoniae* is involved in the regulation of gene expression in response to environmental signals (Bhagirath et al., 2019). These complex pathway networks also need further exploration.

Recent studies on the role of intestinal microbiota in pathogenic gastrointestinal infections allude that these organisms participate in the upregulation or downregulation of bacterial pathogen virulence (Menezes-Garcia et al., 2020; Wang et al., 2020; Cho et al., 2021; Machado Ribeiro et al., 2021; Shil and Chichger, 2021). Upregulation of virulence-related gene expression in *K. pneumoniae* may result in extrahepatic invasion. To further determine the mechanism of IKPLA, we investigated the enrichment of pathogenic genes annotated in PHI-base and the results indicated that the relative abundance of pathogenic genes in IKPLA was significantly higher than that of the KPLA group. Furthermore, a total of 14 pathogenic genes with significant differences in abundance were identified between the two groups. Among them, entA,entC, entF, fepC, fepG, and iucA were the major virulence factors related to iron uptake in *K. pneumoniae* (Yuan et al., 2020); the pilus regulatory gene fimK promotes the virulence of *K. pneumoniae* in murine pneumonia (Rosen et al., 2015; Liu et al., 2022); arcB may mediate resistance against host-derived antimicrobial peptides (Padilla et al., 2010); magA is associated with the virulent hypermucoviscous *K. pneumoniae* strains (Cubero et al., 2016). The relationship between these genes and the invasiveness of KPLA should be verified *via* further research. In addition, KPLA is characterized by solid or multiloculated, which brings difficulties to drainage (Chang et al., 2018). Whether these characteristics are associated with the above-mentioned pathogenic genes is also worth exploring in the future.

Our study has several limitations. First, the number of patients was small. However, our findings could reflect the differences in the microbiota between patients with KPLA and IKPLA. Second, the diagnosis of EMI was based on typical clinical manifestations and imaging features and did not depend on the bacterial culture; thus, the results may be potentially biased. Additionally, although we found that there were significant differences in bacterial composition between the two groups, it was not clear which coexisting bacteria affected the invasiveness of *K. pneumoniae*. Finally, our research lacked experimental verification of the functional pathways and pathogenic genes.

## Conclusions

In conclusion, the bacterial composition of IKPLA was significantly different from that of KPLA. The relative abundance of *unclassified Enterobacteriaceae* and *K. pneumoniae* was high in IKPLA. Microbiological changes in the abscess, activation of the associated metabolic pathways, and the pathogenic gene expression may constitute a new mechanism that regulates the invasiveness of KPLA.

## Data Availability Statement

The datasets presented in this study can be found in online repositories. All the raw sequence reads (FASTQ files) have been deposited in the Sequence Read Archive (SRA) of the National Center for Biotechnology Information (NCBI) with accession number PRJNA782008.

## Ethics Statement

The studies involving human participants were reviewed and approved by the ethics committee of Shengjing Hospital of China Medical University. The patients/participants provided their written informed consent to participate in this study. Written informed consent was obtained from the individual(s) for the publication of any potentially identifiable images or data included in this article.

## Author Contributions

ZZ and ZC designed the study; YG and HW recruited patients and collected clinical data. ZZ, ZC, and HW performed microbiological analyses; ZZ and ZC wrote the paper. YG, HW, and ZL reviewed and edited the manuscript. All authors contributed to the article and approved the submitted version.

## Funding

The study was supported by the National Natural Science Foundation of China (Grant No. 81901856) and the 345 Talent Project in Shengjing Hospital of China Medical University. The funders had no role in the study design, data collection, analysis and interpretation, decision to publish, and preparation of the manuscript.

## Conflict of Interest

The authors declare that the research was conducted in the absence of any commercial or financial relationships that could be construed as a potential conflict of interest.

## Publisher’s Note

All claims expressed in this article are solely those of the authors and do not necessarily represent those of their affiliated organizations, or those of the publisher, the editors and the reviewers. Any product that may be evaluated in this article, or claim that may be made by its manufacturer, is not guaranteed or endorsed by the publisher.

## Supplementary Material

Supplementary Figure 1 Flow chart of patient inclusion.

Supplementary Figure 2 KEGG pathway classification.

Supplementary Table 1 All KEGG Orthology (KO) nesting sites were identified using the KEGG database.

Supplementary Table 2 All key genes from *K. pneumoniae* were annotated using PHI-base.
