# Supplementary figures and images for: Metagenome Analysis of the Bacterial Characteristics in Invasive Klebsiella Pneumoniae Liver Abscesses
Source: Front Cell Infect Microbiol. 2022 Jul 15;12:812542. doi: 10.3389/fcimb.2022.812542 (PMC9334793; doi:10.3389/fcimb.2022.812542)

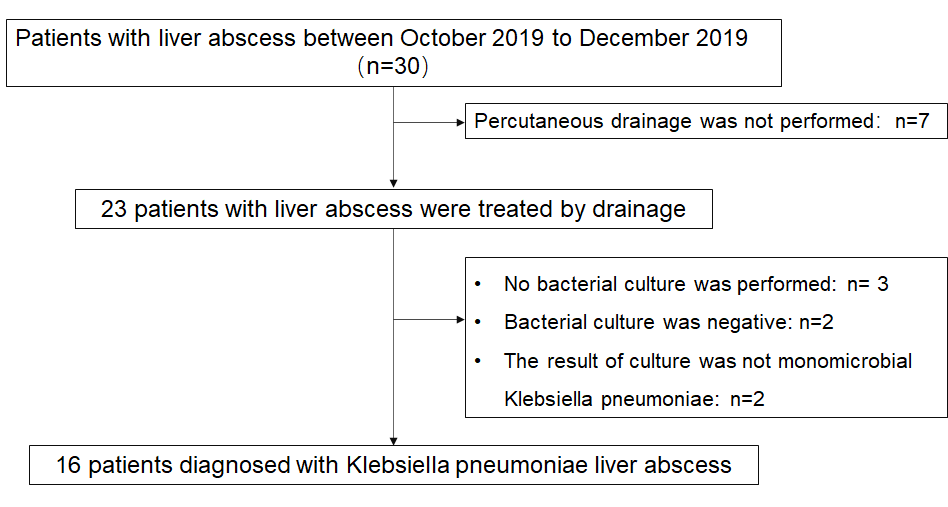

Supplement: Supplementary Figure 1 — Flow chart of patient inclusion. [file Image_1.tif]

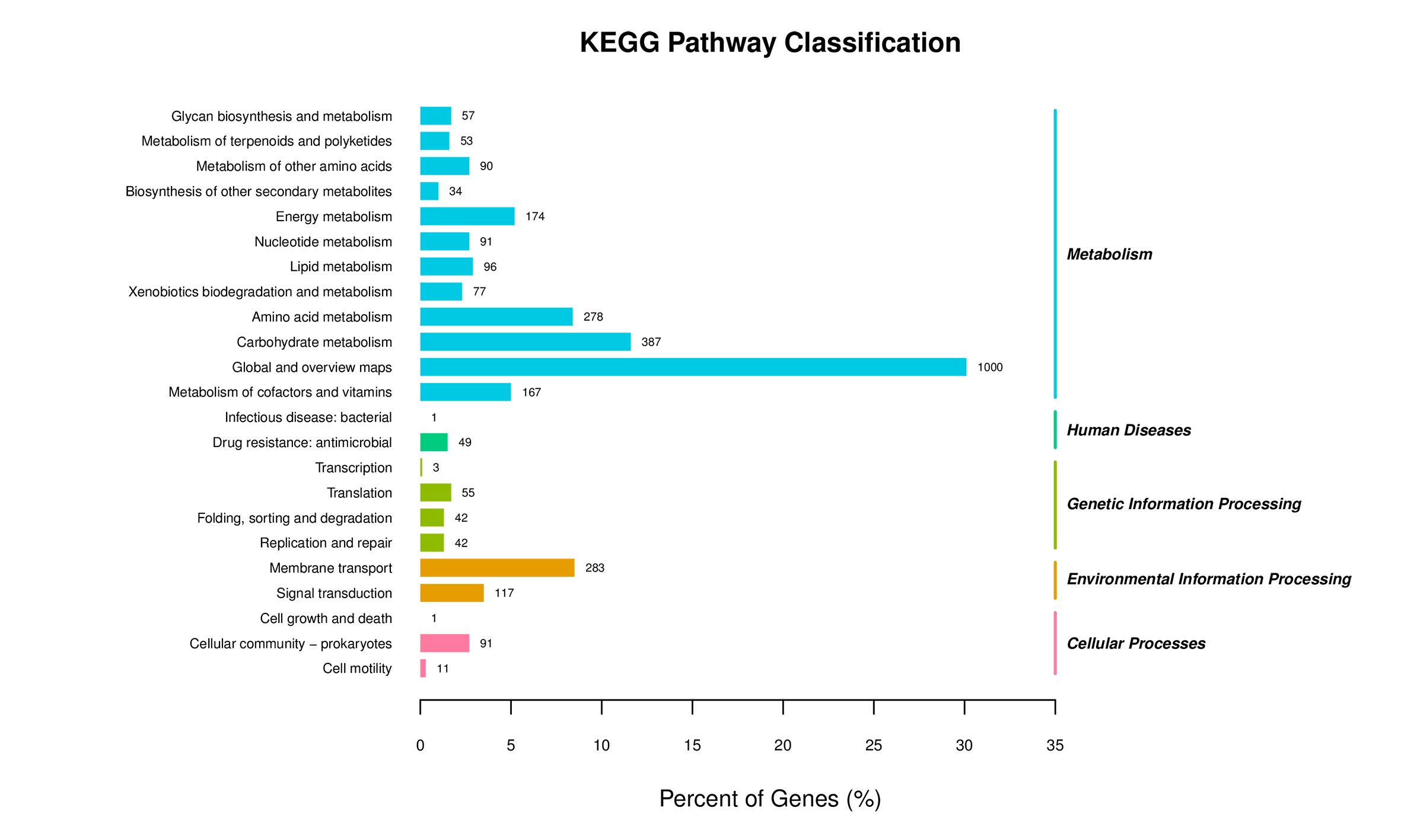

Supplement: Supplementary Figure 2 — KEGG pathway classification. [file Image_2.tif]
